# Mood Regulatory Actions of Active and Sham Nucleus Accumbens Deep Brain Stimulation in Antidepressant Resistant Rats

**DOI:** 10.3389/fnhum.2021.644921

**Published:** 2021-07-19

**Authors:** Rajas P. Kale, Thanh Thanh L. Nguyen, J. Blair Price, Nathanael J. Yates, Ken Walder, Michael Berk, Roy V. Sillitoe, Abbas Z. Kouzani, Susannah J. Tye

**Affiliations:** ^1^Department of Psychiatry and Psychology, Mayo Clinic, Rochester, MN, United States; ^2^School of Engineering, Deakin University, Geelong, VIC, Australia; ^3^Department of Biology and Psychology, Green Mountain College, Poultney, VT, United States; ^4^Department of Neurosurgery Research, Mayo Clinic, Rochester, MN, United States; ^5^Queensland Brain Institute, The University of Queensland, St Lucia, QLD, Australia; ^6^Centre for Molecular and Medical Research, School of Medicine, Deakin University, Waurn Ponds, VIC, Australia; ^7^IMPACT–The Institute for Mental and Physical Health and Clinical Translation, Barwon Health, Deakin University, Geelong, VIC, Australia; ^8^Orygen Youth Health Research Centre, The Department of Psychiatry, University of Melbourne, Parkville, VIC, Australia; ^9^Florey Institute of Neuroscience and Mental Health, University of Melbourne, Parkville, VIC, Australia; ^10^Department of Pathology and Immunology, Department of Neuroscience, Baylor College of Medicine, Houston, TX, United States; ^11^Jan and Dan Duncan Neurological Research Institute, Texas Children’s Hospital, Houston, TX, United States; ^12^Department of Psychiatry, University of Minnesota, Houston, TX, United States; ^13^Department of Molecular Pharmacology and Experimental Therapeutics, Mayo Clinic, Rochester, MN, United States

**Keywords:** deep brain stimualtion, nucleus accumbens, GSK3—glycogen synthase kinase 3, rodent, mTOR—mammalian target of rapamycin, mood, mania, depression

## Abstract

The antidepressant actions of deep brain stimulation (DBS) are associated with progressive neuroadaptations within the mood network, modulated in part, by neurotrophic mechanisms. We investigated the antidepressant-like effects of chronic nucleus accumbens (NAc) DBS and its association with change in glycogen synthase kinase 3 (GSK3) and mammalian target of rapamycin (mTOR) expression in the infralimbic cortex (IL), and the dorsal (dHIP) and ventral (vHIP) subregions of the hippocampus of antidepressant resistant rats. Antidepressant resistance was induced via daily injection of adrenocorticotropic hormone (ACTH; 100 μg/day; 15 days) and confirmed by non-response to tricyclic antidepressant treatment (imipramine, 10 mg/kg). Portable microdevices provided continuous bilateral NAc DBS (130 Hz, 200 μA, 90 μs) for 7 days. A control sham electrode group was included, together with ACTH- and saline-treated control groups. Home cage monitoring, open field, sucrose preference, and, forced swim behavioral tests were performed. Post-mortem levels of GSK3 and mTOR, total and phosphorylated, were determined with Western blot. As previously reported, ACTH treatment blocked the immobility-reducing effects of imipramine in the forced swim test. In contrast, treatment with either active DBS or sham electrode placement in the NAc significantly reduced forced swim immobility time in ACTH-treated animals. This was associated with increased homecage activity in the DBS and sham groups relative to ACTH and saline groups, however, no differences in locomotor activity were observed in the open field test, nor were any group differences seen for sucrose consumption across groups. The antidepressant-like actions of NAc DBS and sham electrode placements were associated with an increase in levels of IL and vHIP phospho-GSK3β and phospho-mTOR, however, no differences in these protein levels were observed in the dHIP region. These data suggest that early response to electrode placement in the NAc, irrespective of whether active DBS or sham, has antidepressant-like effects in the ACTH-model of antidepressant resistance associated with distal upregulation of phospho-GSK3β and phospho-mTOR in the IL and vHIP regions of the mood network.

## Introduction

Deep brain stimulation (DBS) is capable of generating long–lasting neuroadaptations, some of which may contribute to its therapeutic benefits in refractory psychiatric illness (Herrington et al., 2016). Although clinical improvements vary across patients and trials, therapeutic effects are reported to be progressive, suggesting that critical homeostatic networks are engaged and modified by the stimulation over time (Riva-Posse et al., 2018). The nucleus accumbens (NAc) is a common target for DBS therapy across a range of disorders. NAc DBS has been used clinically to alleviate debilitating symptoms of obsessive-compulsive disorder, addiction, depression, Tourette’s syndrome, chronic pain, anorexia nervosa, and autism spectrum disorder with self-injurious behavior (Kuhn et al., 2007; Müller et al., 2009; Goodman et al., 2010; Wang et al., 2013; Ho et al., 2015; Park et al., 2017). In addition to this, the NAc has also been proposed as a potential target for the treatment of anxiety, obesity, binge eating disorder, and schizophrenia (Doucette et al., 2015; Ho et al., 2015; Gault et al., 2018). However, the mechanisms through which chronic NAc DBS can facilitate therapeutic response across distinct treatment-refractory psychiatric conditions are poorly understood. The location and function of the NAc within the broader mesocorticolimbic circuitry may help to explain its seemingly diverse clinical utility in some of the most difficult to treat patient populations.

The NAc represents an important node in the mesocorticolimbic network and plays a critical role in regulating the expression of motivated behaviors, including stress-coping (Eisch et al., 2003; Nestler and Carlezon, 2006; Yadid and Friedman, 2008; Tye and Deisseroth, 2012; Tye et al., 2013). However, beyond this, the NAc serves as a gatekeeper of limbic and cortical information flow through the basal ganglia, governing the neural and behavioral implications of upstream afferent activity (Floresco et al., 2001; Goto and Grace, 2005). Afferents from the prefrontal cortex and limbic system project to the NAc wherein local dopamine signaling functionally selects for predominant cortical vs. limbic activation of efferent projections to the basal ganglia (O’Donnell and Grace, 1995; Grace, 2000). This has important consequences for signal processing and plasticity within this network. The NAc is differentiated into relatively discrete core and shell regions (Zahm, 1999, 2000; Voorn et al., 2004). This is well mapped in the rodent, but similar distinctions have been identified in primate, including human, brains (Meredith et al., 1996). In rats, the shell region receives converging inputs from the basolateral amygdala and ventral subiculum of the hippocampus, while the NAc core receives inputs from the basolateral amygdala and parahippocampal regions (Groenewegen et al., 1999; French and Totterdell, 2003; Voorn et al., 2004). Importantly, while these anatomic and functional differences exist, dopamine signaling within the NAc core has been shown to regulate limbic information flow through both core and shell regions, enabling this region to function as a relay station for selecting and integrating the most relevant input among competing limbic and cortical afferents to modulate network function and behavioral output (Ito and Hayen, 2011).

The progressive nature of DBS therapeutic effects suggests that alterations in neural network function in regions distal to the electrode target may occur. Converging data across distinct antidepressant classes with reported efficacy in treatment-refractory psychiatric disorders, including depression, suggest that glycogen synthase kinase 3 (particularly its beta variant; GSK3β; Luykx et al., 2010; Jope, 2011) and mammalian target of rapamycin (mTOR) signaling (Li et al., 2010; Duman et al., 2014) play important roles in this process, particularly in regions such as the infralimbic cortex (IL) and hippocampus, which are vulnerable to the effects of stress and responsive to the plasticity promoting actions of antidepressants (Wood et al., 2004; Bigio et al., 2016). Both of these signaling proteins rapidly coordinate cellular inflammatory, metabolic, and growth processes to regulate neuroplasticity. In line with this, mTOR and GSK3 signaling has been proposed to play an important role in regulating the recovery of stress–induced dendritic atrophy in the hippocampus and prefrontal cortex (Duman, 2002; Popoli et al., 2002) as well as antidepressant response to lithium augmentation (Walker et al., 2019) and DBS (Kim et al., 2016) in rodent models of treatment resistant depression. Despite well-established direct and indirect projections between the NAc and hippocampus, it remains unclear as to how NAc DBS impacts key mediators of neuroplasticity and neurogenesis within the hippocampus.

To address this, we aimed to determine the antidepressant actions of chronic DBS of the NAc core and its associated impact on hippocampal levels of GSK3β and mTOR (total and phosphorylated) in an established rodent model of tricyclic antidepressant resistance (Kitamura et al., 2002; Müller and Holsboer, 2006; Walker et al., 2013). In this animal model, administration of adrenocorticotropic hormone (ACTH) for 3–14 days, blocks the antidepressant-like effects of tricyclic antidepressants such as imipramine. We validated that 7 days of ACTH treatment blocked the immobility-reducing effects of imipramine and explored the impact of NAc DBS on home cage activity, sucrose consumption, and forced swim immobility time in this model. Previous work in this tricyclic antidepressant-resistant model has demonstrated a depletion of dopamine in the prefrontal cortex tissue following exposure to stress, suggestive of a dysfunctional dopamine system (Walker et al., 2013). Findings from the present study confirm our previous observations (Kim et al., 2016) of NAc DBS-induced antidepressant efficacy and further demonstrates a role for NAc DBS in the modulation of mTOR and GSK3 signaling in the ventral hippocampus. In addition to this, we observed that either active DBS or sham electrode placement in the NAc core normalized observed elevations in sucrose consumption in this model, while also being associated with an overall increase in homecage activity across the course of the experiment.

## Materials and Methods

### Animals

**Figure 1:**
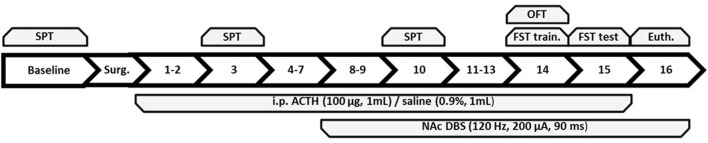
Summary of the experimental timeline with each number corresponding to the day following baseline and surgery. Behavioral tests administered to all groups are on top of the figure and treatments are below. SPT, sucrose preference test; OFT, open field test; FST, forced swim test; train., training; Euth., euthanasia; ACTH, adrenocorticotropic hormone; NAc DBS, nucleus accumbens deep brain stimulation.

All procedures were reviewed and approved by Mayo Clinic’s Institutional Animal Care and Use Committee (IACUC) and the University of Queensland’s Animal Ethics Committee (AEC). Efforts were made to minimize animal usage. Six cohorts totaling 85 male Wistar rats (Harlan, IN, USA; Animal Resource Centre, WA, Australia) were individually housed with water and chow available *ad libitum*. Seventy-seven animals were used in the final analysis, accounting for the loss of eight animals in total [4 lost as behavioral outliers (2 DBS, 1 ADR, 1 SAL), four due to electrode or device damage (3 DBS and 1 sham group)]. The animal vivarium was under a 12:12 h light:dark cycle with lights on at 7:00 AM. Each cage was fitted with an infrared motion sensor to measure homecage activity, which was streamed to a data acquisition box (Actimetrics, IL, USA). Activity counts were recorded continuously throughout the experiment. The experimental timeline, including treatments and behavioral tests, is described in Figure 1.

The experiment was divided into three phases: week 1 (days −6 to 0; baseline and surgery period), week 2 (days 1–7), and week 3 (days 8–15). Animals were euthanized 1 h following exposure to the FST. ACTH injections were administered across days 1–15, and DBS occurred on days 8–16 up until euthanasia. Animals were divided into three study groups: (1) validation of antidepressant-resistance following 7 days of ACTH treatment; (2) validation of antidepressant resistance following chronic tricyclic antidepressant resistance during week 3 in ACTH-treated rats; and (3) mood regulatory actions of chronic NAc DBS in ACTH-treated antidepressant-resistant rats.

#### Validation of Antidepressant-Resistance at Day 7

In this experiment, animals were divided into four groups and treated either with saline (0.9%) or ACTH (100 μg) each day via intraperitoneal (i.p.) injection. Animals underwent imipramine (10 mg/kg) or control saline (0.9%) treatment on day 7, 1 h before the FST.

#### Validation of Antidepressant Resistance at Day 16

In this experiment, animals were divided into four groups and treated either with saline (0.9%) or ACTH (100 μg) each day via intraperitoneal (i.p.) injection. Animals underwent imipramine (10 mg/kg) or control saline (0.9%) treatment over the course of days 8–16.

#### Antidepressant Actions of Chronic NAc DBS in the Antidepressant-Resistant Rodent Model

In this experiment, animals were divided into four groups and treated either with saline (0.9%) or ACTH (100 μg). ACTH-treated animals then received in active or sham DBS over the course of days 8–16. This experimental design addresses our overarching research question of elucidating mechanisms of DBS in antidepressant-resistant animals.

Treatments administered to each group are described in Table 1; four groups: DBS (*n* = 10), SHAM (*n* = 10), ADR (*n* = 8) and SAL (*n* = 9).

**Table 1:** Group treatments.

Groups	Treatment	NAc Electrodes	DBS
DBS	ACTH	Yes	Yes
SHAM	ACTH	Yes	No
ADR	ACTH	No	No
SAL	Saline	No	No

### Surgery

DBS and SHAM groups were bilaterally implanted with twisted bipolar electrodes (PlasticsOne, VA, USA) through stereotactic surgery into the NAc core using the Bregma (flat skull) as coordinate reference (A-P: +1.5 mm; M-L: ±1.5 mm; D-V: −7.0 mm; Paxinos and Watson, 2005). Animals received anesthesia through inhalation of isoflurane (3.0% induction, 1.5% maintenance) during surgery and were visually monitored through breathing and reflexes. Cranial screws were affixed to the skull and anchored with dental cement, which sealed the burr holes and held the electrodes in place. All tools and equipment were sterilized in 70% ethanol between individual surgeries and autoclaved between surgery days to reduce infection risk. Animals were then allowed to recover for 3 days in homecages and checked daily for any movement complications and proper weight gain.

### Injections

All animals received daily 1 ml intraperitoneal injections of ACTH (AnaSpec, CA, USA) at 100 μg/ml (with the exception of the SAL group, which received 1 ml 0.9% saline injections) at approximately 4 h into the light cycle. This corresponds to the circadian nadir and the lowest plasma corticosterone levels in rats (Watts et al., 2004). Injections occurred every day from day 1 to day 15 (inclusive). Animals did not receive injections on the day of euthanasia.

### Deep Brain Stimulation Device

DBS devices were soldered to electrode cords (Plastics One, VA, USA) that screw onto the implanted electrode. Each device was tested under an oscilloscope to ensure a complete and correct pulse (130 Hz, 200 μA, 90 μs). The single-piece back mountable DBS devices were designed to produce monophasic constant current pulses for over 12 days with a single battery while incorporating passive charge balancing (Kouzani et al., 2013). Two DBS devices were enclosed into a pouch and affixed to the back of a rat vest (Harvard Apparatus, MA, USA) to deliver continuous stimulation for animals in the DBS group over days 8–16 (week 3). SHAM animals wore the vests during this time.

### Sucrose Preference Test

The 2-bottle choice sucrose preference test (SPT) screens for sucrose-associated carbohydrate preference, a behavioral model associated with depression. Other SPT methods may also screen for anhedonia in cases where the animal has to work to obtain the sucrose reward (Felger et al., 2013), but in this case, the two-bottle choice offers equal effort for either bottle and does not necessarily detect anhedonia. Changes in preference over each experimental phase are used to determine treatment-based sensitivity to rewarding stimuli in the form of carbohydrate consumption. The SPT consists of three recorded preference tests, and one initial exposure session 2 days prior to the first recorded test. During the exposure session, animals were deprived of water for 16 h prior to the test. The SPT began 4 h after the start of the light cycle. Preference was determined using a 2-bottle choice, with one bottle containing water and the other containing 1% sucrose solution. For the SPT’s the same procedure was followed with the addition of measuring the weights of both bottle types before and after administration to record the amount consumed by each animal. The left and right side for which each bottle is placed were switched between each animal and between each SPT trial to avoid place preference. A total of one exposure and three SPT’s were run, where the exposure session and first SPT were performed during the baseline period (day −6), and the subsequent two SPT’s performed on experimental days −3, 3, and 10.

### Open Field Test

The open field test (OFT) quantifies psychomotor and anxiety-like behavior in a novel low-stress environment by measuring total distance moved, the velocity of movement, time spent in the center region (high-anxiety), and time spent in the periphery (low anxiety) region. This distinction between low and high anxiety regions is enhanced when a bright light illuminates the central area. The current protocol incorporated lighting throughout the full arena, thereby limiting this distinction and consequent anxiogenic nature of the central area. The apparatus is a square-shaped box (59.7 × 59.7 × 43.2 cm) throughout which the animal is free to move. Rat vests and DBS devices were unplugged and removed before initiating OFT in DBS and sham groups. Thus, all animals underwent this trial without a vest, facilitating direct comparison of behavioral data across groups. Prior work suggests this brief lapse in DBS application does not impact behavioral effects in this test (Kim et al., 2016). Animals were individually placed in the center of the apparatus floor for 10 min prior to injections. Video recording of the animal’s movements was analyzed using TopScan software (CleverSys Inc, VA, USA) for the first 5 min after placing the animal in the center. The mean time spent in the central region, as well as distance traveled, was compared among groups for anxiety-like behavior and psychomotor activity. The OFT was performed on Day 14 prior to treatment injections.

### Forced Swim Test

The forced swim test (FST) is a measure of psychomotor behavior and a screen of antidepressant efficacy under a high-stress environment (Porsolt et al., 1977). Animals were placed into acrylic cylinders (height = 47 cm, diameter = 19 cm) with 25°C tap water filled to 28 cm from the base. A 15-min FST training session was performed 2 h after OFT and injections on Day 14, and the 6-min recorded test FST session was performed on Day 15, again 2 h after injections. DBS devices, which are not waterproof, and vests remained removed from previous OFT for FST training session, and were unplugged and removed prior to recorded test FST session. Prior work suggests this brief lapse in DBS application does not impact the behavioral efficacy of DBS in this test (Kim et al., 2016). Video recording of the test session was analyzed through hand-scoring to measure the time animal spent performing active (swimming, climbing, diving) vs. passive (immobility) coping behaviors during the session. The latency to the first 2-s immobility interval was also recorded for each animal.

### Euthanasia and Tissue Collection

Animals were euthanized via anesthetic overdose (FatalPlus; Vortech Pharmaceuticals, MI, USA). Whole brains were harvested and stored at −80°C. During tissue collections, brains were slightly thawed on a cooling block (BioCision, CA, USA) for tissue collection. One mm coronal slices were cut at regions of interest and a 1 mm diameter biopsy punch collected the samples to be stored at −80°C.

### Protein Analysis

Ventral hippocampus (vHipp) and dorsal hippocampus (dHipp) concentrations of GSK3β, p-GSK3β, mTOR, and p-mTOR at the time of euthanasia were determined through western blot. Tissue samples were lysed in radioimmunoprecipitation assay (RiPA) lysis buffer. The total protein concentration was determined by Bradford assay (BioRAD, CA, USA). Twenty microgram of protein lysate were subjected to sodium dodecyl sulfate polyacrylamide gel electrophoresis (SDS-PAGE) and then transferred to polyvinylidene fluoride (PVDF) membrane (Immobilon-P). Membranes were blocked for 2 h in tris buffered saline solution with 0.1% Tween 20 (TBST) and 5% milk. Membranes were incubated with a 1:1,000 dilution of GSK3β, p-GSK3β (Ser21/9), mTOR, and p-mTOR (Ser2448) primary antibodies produced in rabbit (Cell Signaling Technology, MA, USA) in PBST overnight. The membranes were washed three times (10 min each) the following day with phosphate-buffered saline with 0.1% Tween 20 (PBST), and incubated with a 1:2,000 dilution of anti-rabbit HRP-linked secondary antibody (Cell Signaling Technology, MA, USA) in PBST for 1 h. Blots were again washed three times (10 min each) with PBST before being exposed to enhanced chemiluminescence (ECL) substrate (Thermo Scientific, Rockford, IL, USA). β-Actin was determined using β-Actin primary antibody produced in mouse (Sigma Aldrich, MO, USA) with a dilution of 1:10,000 in TBST, and anti-mouse HRP-linked secondary antibody (Cell Signaling Technology, MA, USA) with a dilution of 1:5,000 in PBST. The resulting bands were measured using densitometric analysis on a Bio-Rad ChemiDoc^TM^ imaging system. Readings on each gel were first normalized to β-Actin, and then to the average protein concentration of saline animals on each gel.

### Histology

The remaining portion of the brain was embedding using Cryo-M-Bed (A-M Systems 527738). Coronal sections of 6 μm were taken around NAc. The sections were fixed using ice-cold acetone for 10 min, followed by 10 min of drying time.

**Figure 2:**
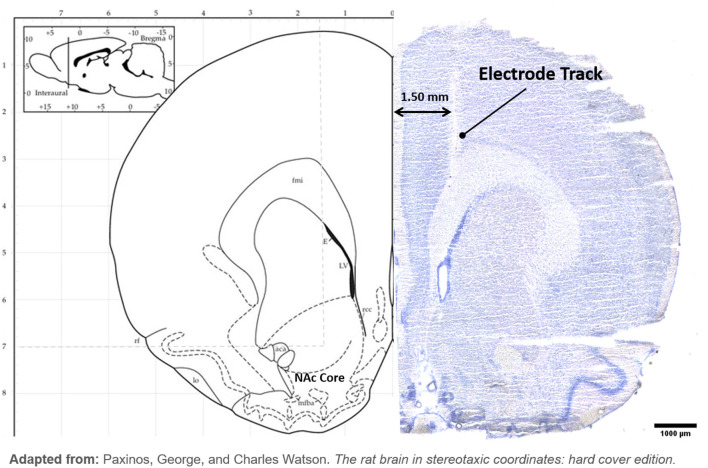
Hybrid image with the left hemisphere of atlas brain (Paxinos and Watson, 2005) at NAc and example of H&E stained brain. (Left): Electrode tip placements are displayed with a black circle in the left hemisphere only for each DBS animal. (Right): DBS electrode tract is imaged terminating in NAc.

H&E Staining: after fixation and drying, the slides were then incubated in phosphate-buffered saline for 10 min, followed by 30-s incubation in hematoxylin stain, then rinsed under running water for 5 min. The following dips were done: Blueing four dips, water 10 dips, 95% Ethanol four dips, Eosin six dips, 50% Ethanol 12 dips, 70% Ethanol 12 dips, 95% Ethanol 12 dips, Absolute Ethanol 12 dips, and Xylene 12 dips. Slides were allowed to completely dry before preserving in Vecta-mount (Vector Laboratories LTD, CA, USA) and coverslipping. Example of DBS tract and all electrode positions were recorded (Figure 2).

### Statistical Analyses

Animals were excluded from the study if the animal was terminated before the end of the experiment or if corresponding protein data was unavailable. Additionally, data outliers were identified as values greater than two standard deviations above or below the mean of the group data and were excluded from that dataset. D’Agostino and Pearson omnibus normality test was then performed. If all groups passed this normality test in a dataset (alpha = 0.05), parametric one-way analysis of variance (ANOVA) was performed to determine the overall main effect (*p* < 0.05) or trending effect (*p* < 0.1). If no effect was demonstrated, no further actions were taken. If an effect was demonstrated (*p* < 0.1), Sidak’s *post hoc* multiple comparisons tests were performed to determine group differences. Repeated measures ANOVA was performed for SPT datasets followed with Tukey’s *post hoc* multiple comparisons tests if an effect was demonstrated. If any groups failed the normality test in a dataset (vHipp GSK3β and p-GSK3β protein levels), non-parametric Kruskal-Wallis H test with Dunn’s *post hoc* multiple comparisons test was performed to determine group differences. Group comparisons were only performed between groups that differed by exactly one treatment (i.e., DBS-SHAM, DBS-ADR, SHAM-ADR, and ADR-SAL). If the sample size of any group was too low for the D’Agostino and Pearson omnibus normality test, the Shapiro-Wilk normality test was performed for that group to determine normality. All analyses and graphs were created using GraphPad Prism 6.0.

## Results

### Forced Swim Test

**Figure 3:**
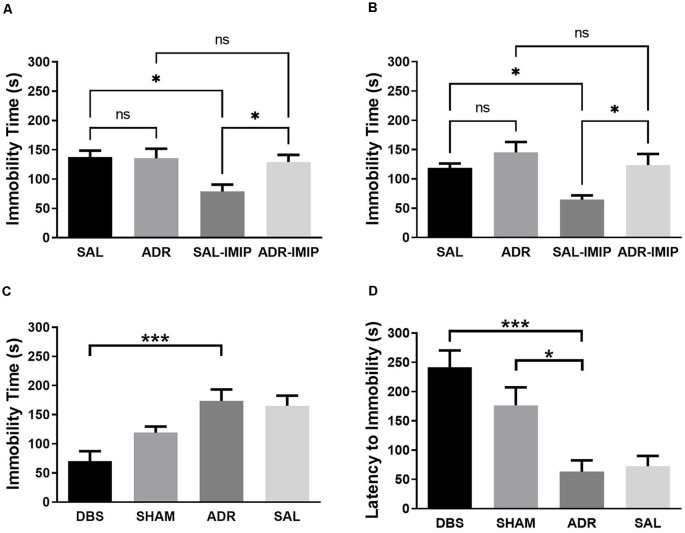
Forced swim test total immobility time when antidepressant resistant (ADR) and saline control (SAL) animals are treated with imipramine (IMIP) at **(A)** 7 days and **(B)** 16 days into the ACTH treatment protocol, demonstrating validity of the ADR phenotype; and **(C)** immobility and **(D)** latency to first immobility following DBS or SHAM treatment. Data shown in mean seconds standard error. Significance is illustrated as *p < 0.05; ***p < 0.001; ns, not significant.

Imipramine effectively reduced immobility time in animals pretreated with saline, but not ACTH at each time point (7 days and 16 days; Figures 3A and 3B). NAc DBS significantly lowered FST immobility time (*p* < 0.001). This effect was not observed with sham treatment [Figure 3C; *p* < 0.001, *F*_(DFn,Dfd)_ = 8.740 (3, 34)]. Latency to first immobility of 2-s duration was significantly increased by NAc DBS (*p* < 0.001) and sham (*p* = 0.018) treatment [Figure 3D; *p* < 0.001, *F*_(DFn,Dfd)_ = 11.01 (3, 33)].

### Open Field Test

**Figure 4:**
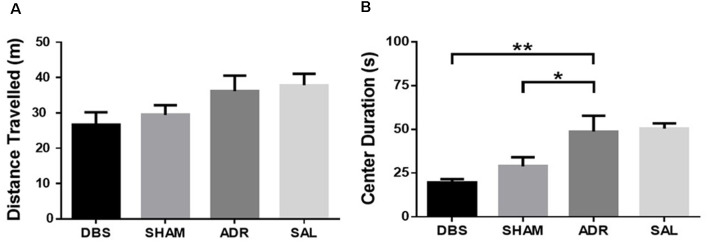
Open field test **(A)** distance traveled and **(B)** time spent in the center region. Data shown in mean seconds ± standard error. Significance is illustrated as *p < 0.05; **p < 0.01.

No differences were observed in the total distance traveled during OFT. These results validate FST measures of antidepressant-like effects [Figure 4A; *p* = 0.081, *F*_(DFn,Dfd)_ = 2.446 (3, 34)]. DBS (*p* = 0.002) and sham (*p* = 0.033) treatment significantly lowered the total duration spent in the center region of the OFT apparatus [Figure 4B; *p* < 0.001, *F*_(DFn,Dfd)_ = 8.615 (3, 33)].

### Activity Profiles

The activity was measured with an infrared beam motion sensor attached to each cage. Activity across the baseline period (days −6 to 0) was averaged, excluding surgery and recovery days. Week 2 activity was averaged from days 2 through 7 and week 3 was averaged from days 9 through 14. Activity profiles are represented as percent change to minimize variabilities between individual sensor sensitivities.

**Figure 5:**
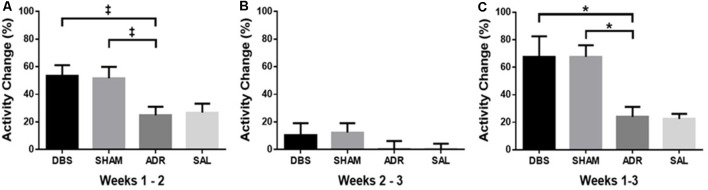
Percent change in homecage activity counts between **(A)** pretreatment baseline and the first week of ACTH treatment (days 1–7), **(B)** first week of ACTH treatment (days 1–7), and second week of ACTH treatment with DBS (days 8–15), **(C)** pretreatment baseline and second week of ACTH treatment with DBS (days 8–15). Data shown in mean percentage change of activity counts per day ± standard error. Significance is illustrated as *p < 0.05. Trends illustrated as ^‡^*p* < 0.1.

An increase in activity was observed in both the DBS (*p* = 0.051) and SHAM (*p* = 0.071) groups when comparing the average of baseline days (days −6 to 0) relative to days 1–7. This activity increase corresponds with electrode implantation in these groups [Figure 5A; *p* < 0.05, *F*_(DFn,Dfd)_ = 4.299 (3, 34)]. No difference in activity was detected between weeks 2 and 3 [days 1–7 relative to days 8–15; Figure 5B; *p* = 0.443, *F*_(DFn,Dfd)_ = 0.917 (3, 34)]. Overall, activity across all 3 weeks (days −6 to 15) was significantly higher in DBS (*p* < 0.05) and SHAM (*p* < 0.05) groups [Figure 5C; *p* < 0.01, *F*_(DFn,Dfd)_ = 6.428 (3, 33)].

### Sucrose Preference Test

**Figure 6:**
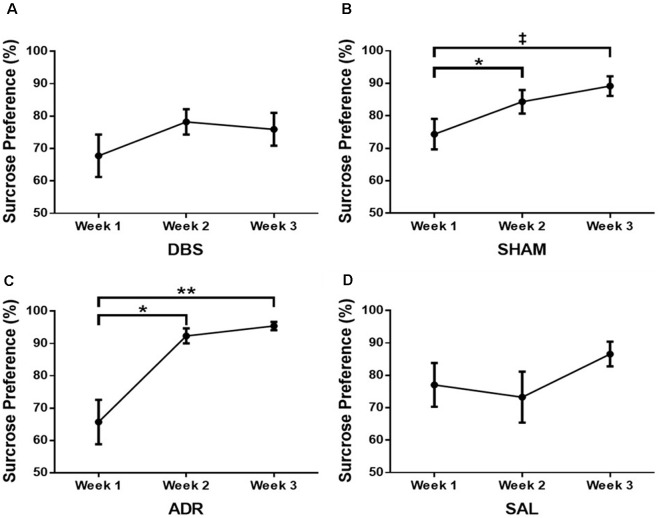
Sucrose preference test at each week for **(A)** DBS, **(B)** sham, **(C)** ACTH, **(D)** saline groups. Data are shown as mean ± standard error. Significance is illustrated on graphs by *p < 0.05; **p < 0.01. Trends illustrated as ^‡^*p* < 0.1.

No difference was observed in sucrose preference changes for the DBS group [Figure 6A; *p* = 0.3249, *F*_(DFn,Dfd)_ = 1.168 (1.498, 13.48)]. Increased sucrose preference was demonstrated by day 10 for the SHAM (*p* < 0.05) and ADR (*p* < 0.05) groups [Figure 6B; *p* < 0.05, *F*_(DFn,Dfd)_ = 5.355 (1.575, 12.60; Figure 6C; *p* < 0.01, *F*_(DFn,Dfd)_ = 16.03 (1.205, 8.434)]. No difference was observed in sucrose preference changes for the SAL group [Figure 6D; *p* = 0.251, *F*_(DFn,Dfd)_ = 1.517 (1.359, 12.23)].

### Protein Analyses

**Figure 7:**
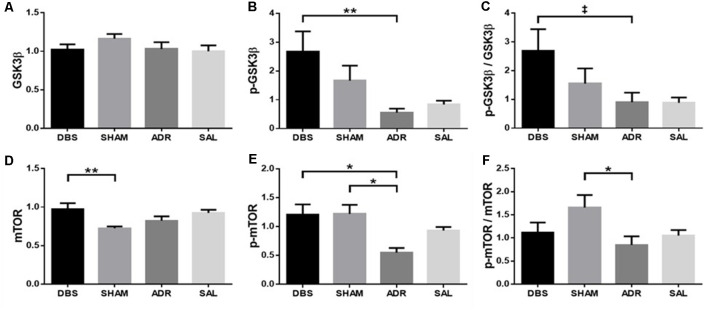
vHipp protein expression levels of **(A)** GSK3β, **(B)** p-GSK3β, **(C)** p-GSK3β/GSK3β, **(D)** mTOR, **(E)** p-mTOR, **(F)** p-mTOR/mTOR. Data are shown as mean concentration standardized to β-actin and normalized to SAL controls for each gel ± standard error. Significance is illustrated on graphs by *p < 0.05; **p < 0.01. Trends illustrated as ^‡^*p* < 0.1.

vHipp protein levels (GSK3β, p-GSK3β, mTOR, p-mTOR) were characterized at the time of euthanasia (day 16). No significant differences were detected in vHipp GSK3β levels (Figure 7A; *p* = 0.3265, *H* = 3.456). NAc DBS elevated p-GSK3β levels (*p* < 0.01) as well as p-GSK3β/GSK3β levels [*p* = 0.086; Figure 7B; *p* = 0.0151, *H* = 10.44; Figure 7C; *p* = 0.058, *F*_(DFn,Dfd)_ = 2.766 (3, 32)]. NAc DBS elevated mTOR levels compared to SHAM animals [*p* < 0.01; protect; Figure 7D; *p* < 0.01, *F*_(DFn,Dfd)_ = 4.661 (3, 31)]. Both NAc DBS (*p* < 0.05) and sham (*p* < 0.05) treatments elevated p-mTOR levels [Figure 7E; *p* < 0.05, *F*_(DFn,Dfd)_ = 4.380 (3, 32)]. Sham treatment elevated p-mTOR/mTOR levels [*p* < 0.05; Figure 7F; *p* = 0.057, *F*_(DFn,Dfd)_ = 2.773 (3, 33)].

**Figure 8:**
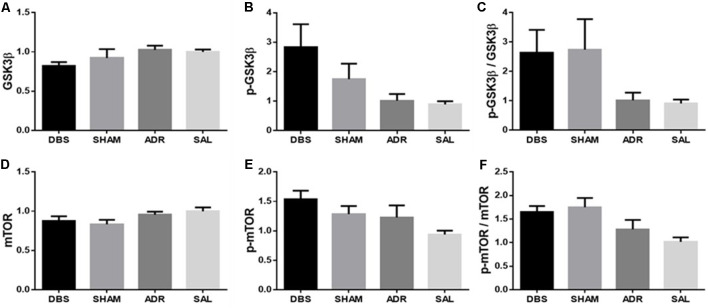
dHipp protein expression levels of **(A)** GSK3β, **(B)** p-GSK3β, **(C)** p-GSK3β/GSK3β, **(D)** mTOR, **(E)** p-mTOR, **(F)** p-mTOR/mTOR. Data are shown as mean concentration standardized to β-actin and normalized to SAL controls for each gel ± standard error.

dHipp protein levels were also characterized, however no significant effects were detected between groups. A significant effect was detected in p-GSK3β levels [Figure 8B; *p* < 0.05, *F*_(DFn,Dfd)_ = 2.913 (3, 32)], but neither Sidak’s *post hoc* multiple comparisons tests nor *t*-tests with Bonferroni corrections between compared groups revealed significant or trending differences [Figure 8A; *p* = 0.210, *F*_(DFn,Dfd)_ = 1.593 (3, 33); Figure 8C; *p* = 0.155, *F*_(DFn,Dfd)_ = 1.874 (3, 31); Figure 8D; *p* = 0.125, *F*_(DFn,Dfd)_ = 2.058 (3, 33); Figure 8E; *p* < 0.05, *F*_(DFn,Dfd)_ = 3.044 (3, 33); Figure 8F; *p* < 0.01, *F*_(DFn,Dfd)_ = 4.813 (3, 33)].

## Discussion

This study provides further support for the antidepressant actions of NAc DBS using reduced immobility in the forced swim test as our primary proxy of antidepressant efficacy. ACTH-pretreated animals treated with NAc DBS remained immobile for significantly less time relative to control ACTH-treated rats (ADR). No significant difference was observed in immobility time between the SHAM group and controls. Both DBS and SHAM groups demonstrated elevated latency to immobility, a secondary indicator of antidepressant-like behavioral effects. When coupled with reduced total immobility behavior, as observed in the DBS-treated group, this provides further evidence for the potential antidepressant actions of the intervention. Given this pairing of total immobility and latency to immobility did not occur in the SHAM group, this increase in latency alone cannot be interpreted as a robust antidepressant response. However, the visual (non-significant) trend towards reduced immobility time, coupled with significant increases in latency to immobility suggests that the effects of electrode implantion, potentially moderated by inflammatory factors, persisted until behavioral tests were carried out as previously reported by others (Perez-Caballero et al., 2014). In addition to these effects in the FST, we observed an increase in homecage activity in both DBS and SHAM groups throughout the experiment. In contrast, increased psychomotor activity was not observed in the novel environment of the OFT. Although it remains unclear why this distinction was observed, it does serve to validate results from the forced swim antidepressant screening efficacy as measures of stress coping, negating the potential for confounds due to locomotor hyperactivity. We further observed an increase in sucrose consumption in ACTH and SHAM animals, relative to DBS and SAL groups as well as elevated levels of pGSK3β and pmTOR in the ventral striatum of DBS (pGSK3 and pmTOR) treated animals. In contrast, elevated levels of pmTOR only were in the vHIP of the SHAM group.

The marked reduction in passive coping behaviors (i.e., immobility in the FST) suggests that both active and sham NAc DBS exerted an antidepressant-like effect in this behavioral assay. This antidepressant effect was validated by the lack of significant change in locomotor activity in the OFT; implying the FST effectively measures antidepressant-like increases in active coping behavior and not stress-related hyperactivity. It is also important to note that both ACTH and SAL animals exhibited elevated levels of immobility relative to non-stressed pair-housed control animals, with no significant differences observed between the ACTH and SAL groups. While this baseline comparison shows no difference between the ACTH and SAL groups, they were clearly distinguished by their response to imipramine following either 7 days or 16 days of their respective daily treatments. Collectively, these data confirm that, under the conditions of the present study where both groups were socially isolated and received daily i.p. injections, the ACTH- and SAL-treatment protocols elicited a tricyclic antidepressant-resistant and responsive depressed phenotype, respectively. This underscores the limitations of the FST behavioral model as one of the quantifying mechanisms of antidepressant-response, rather than phenotyping nuanced features of depression-like behavior (Nestler and Hyman, 2010). The findings in this study, therefore, contribute towards elucidating the antidepressant mechanisms of action of DBS in an animal model of ADR.

Previously we have shown that NAc electrode implantation (active or sham) induced a state of hyperactivity in a subgroup of rats (Kim et al., 2016). Here, while not as robust an effect as previously reported, the observed increase in homecage activity in ACTH-treated rats underscores the potential for disruption of the NAc via electrode implantation to enhance behavioral activity. Stress associated with daily i.p. injections appeared to increase activity in all groups when compared with pretreatment baseline levels. On top of this, electrode implantation was also associated with an elevation in homecage activity when compared with baseline averages. These changes in homecage activity may reflect changes in mesoaccumbens dopamine signaling resulting from the disruption, which is well-established to affect locomotor activity. Similarly, dopamine dysregulation is inferred by elevations of carbohydrate consumption from ADR and SHAM groups during the SPT. Indeed, the mesolimbic dopamine pathway acts as an important regulator for feeding behavior and food craving (Berthoud et al., 2011), and modulating these pathways through NAc DBS countered this ACTH-mediated increase in sucrose consumption. As suggested by our previous observations of hyperactivity in a subset of DBS and SHAM animals pretreated with ACTH, but not saline (Kim et al., 2016), this may be particularly relevant in models where dopamine dysfunction is an underlying component of the pathophysiology (Walker et al., 2013). Interestingly, in clinical trials for anorexia nervosa, ablation or active DBS of the NAc was therapeutically effective through 1 year, improving basic vital signs and body mass index (Wang et al., 2013). In addition to these critical metrics, associated improvements on scores of depression, anxiety and obsessive-compulsive disorder, social functioning, and quality of life were also observed (Wang et al., 2013).

In addition to this potential impact of electrode implantation on a dysfunctional dopamine system in these outcomes, it is important to note that antidepressant actions of electrode impanation have been reported previously in rodents and shown to be mediated by an inflammatory response to acute tissue trauma (Perez-Caballero et al., 2014). Retrospective, correlative data from human patients further suggested that anti-inflammatory treatment at the time of DBS surgery was noted to be associated with reduced clinical efficacy in a small cohort of patients (Perez-Caballero et al., 2014). These authors demonstrated an acute antidepressant-like effect of electrode implantation into the infralimbic cortex. This effect was temporally correlated with an increase of glial-fibrillary-acidic-protein expression and other inflammatory mediators, suggesting it was due to regional inflammation. In support of this, the effects of sham implantation were blocked by anti-inflammatory drugs. These findings indicate that network-wide adaptations occur in response to active DBS or SHAM electrode placement to impact behavior, including antidepressant responses.

In the current study, we observed some behavioral responses to electrode implantation. However, the progressive timeline of these measures means that any potential therapeutic impact of inflammation would have likely reduced over time. This may explain the more robust behavioral response to active DBS in the FST, as well as on post-mortem tissue levels of hippocampal cell signaling proteins GSK3β and mTOR. Alterations in GSK3 function within the CNS are associated with depressive-like and mania-like behavior in rats and can play an important role in mood stabilization and antidepressant efficacy clinically (Jope, 2011). GSK3 also contributes to the regulation of anti-inflammatory responses that can exacerbate depression (Martin and Leibovich, 2005), and long–term disruption of its activity within the hippocampus is associated with cognitive impairment and neurodegeneration over time (Bradley et al., 2012). Inhibition of GSK3 by phosphorylation may therefore provide an indicator of changes in cellular pathways critical for coordinating antidepressant responses. Our study suggests the efficacy of NAc DBS in treatment-resistant depression as it significantly increased p-GSK3β levels in the vHipp, and this effect was not confounded by sham electrode implantation. The signaling cascade of mTOR is responsible for numerous cell functions such as synaptic plasticity, mitochondrial metabolism, and neurogenesis. For example, activation of mTOR is critical for the therapeutic response of antidepressants due to its involvement in the synthesis of downstream synaptic protein (Duman et al., 2014). Activation of mTOR by phosphorylation at Serine 2448 can serve as an indicator of enhanced neurogenesis that is partly responsible for the antidepressant effect. Our study suggests that activated mTOR levels in the vHipp were increased in both the DBS and SHAM groups, which means a therapeutic response was observed in this treatment-resistance model but a specific mechanism remains unknown. The antidepressant effect of electrode implantation regardless of electrical stimulation has been observed in other studies, and it was suggested that early anti-depressive response in sham treatment is due to inflammation (Perez-Caballero et al., 2014). Finally, treatment effects on signaling proteins were observed in the vHipp, while treatment effects were not observed in dHipp. Because the vHipp has been linked to emotional behavior and neuroendocrine stress-related regulation, and dHipp more to learning and memory (Tanti and Belzung, 2013), an enhanced neurogenesis effect by DBS within the vHipp is specifically relevant to the intended antidepressant effects. Furthermore, a recent study showed that neurogenesis in the hippocampus is not only necessary but also sufficient to decrease anxiety and depressive-like behaviors in rats (Hill et al., 2015), suggesting that NAc DBS, through its molecular regulation in the hippocampus, is a promising treatment strategy for refractory depression.

This study offers a unique approach to studying DBS in rats using a model of treatment-resistant depression in that it incorporates continuous bilateral stimulation through an untethered device affixed to the rat to maximize portability. Many previous studies accomplished intermittent unilateral stimulation for up to a few hours (Gersner et al., 2010; Schmuckermair et al., 2013; Hamani et al., 2014). Future studies with portable optogenetic microdevices (Kale et al., 2015) could offer precise regional and cell-specific stimulation to more effectively delineate the role of inflammatory responses in the early antidepressant actions of DBS. However, it is also important to acknowledge our choice not to perform DBS and SHAM treatment in control (saline-treated) animals limits our ability to confirm that the effects observed in the current study are unique to the ADR model. Our prior work has shown that DBS has antidepressant actions in both SAL and ACTH treated groups (Kim et al., 2016), however, we cannot be sure if the early inflammatory response implicated in the antidepressant actions of active and sham DBS observed herein are moderated by stress hormone pretreatment. Future comprehensive biochemical and ‘omic’ analyses could further provide insight into causal mechanisms of antidepressant action and biomarkers of DBS response efficacy.

## Conclusion

NAc DBS appears to have effective disease-modifying actions for refractory psychiatric indications. Our observations of the immobility-reducing effects of NAc DBS in the ACTH-treated model of ADR underscore its potential utility in treatment-resistant conditions. Observations of elevated homecage activity and a reversal in sucrose consumption behaviors relative to ACTH-treated animals underscore the need for further investigation of the effects of NAc DBS or SHAM electrode placement on dopamine system function. Given DBS might provide antidepressant efficacy in treatment-refractory psychiatric disorders, additional animal studies in translationally relevant ADR models are necessary to investigate its critical mechanisms of antidepressant action. Comparison of acute and chronic effects of DBS on key behavioral and molecular metrics of antidepressant efficacy in rodent models of ADR can help us to better understand the neuromodulatory mechanisms of this treatment in refractory conditions. Such work would ideally have groups receiving chronic DBS continuously throughout the experiments, with comparative control animals wearing vests throughout the study to enable direct comparison across homecage and behavioral test conditions. Further understanding of the molecular mechanisms through which NAc DBS and/or SHAM electrode placement promotes distal changes within the mesocorticolimbic network could provide new opportunities for alternate or augmented therapeutic strategies for psychiatric DBS.

## Data Availability Statement

The raw data supporting the conclusions of this article will be made available by the authors, without undue reservation.

## Ethics Statement

The animal study was reviewed and approved by Mayo Clinic Institutional Animal Care and Use Committee and the University of Queensland Animal Ethics Committee.

## Author Contributions

All authors contributed to conception and design of the work. RK, TN, JP, and NY were involved in the acquisition and analysis of the data. RK, TN, JP, NY, and ST analyzed the data and prepared a draft of the manuscript. All authors contributed to study design, data review, preparation of the manuscript, and approved the submitted version.

## Conflict of Interest

RK and JP were supported by a Deakin University Postgraduate Award. TN was supported by a Mayo Clinic Graduate Student Scholarship. MB was supported by an NHMRC Senior Principal Research Fellowship (GNT1059660) and has received Grant/Research Support from the NIH, Cooperative Research Centre, Simons Autism Foundation, Cancer Council of Victoria, Stanley Medical Research Foundation, MBF, NHMRC, Beyond Blue, Rotary Health, Geelong Medical Research Foundation, Bristol Myers Squibb, Eli Lilly, Glaxo SmithKline, Meat and Livestock Board, Organon, Novartis, Mayne Pharma, Servier, Woolworths, Avant and the Harry Windsor Foundation, and has been a speaker for Astra Zeneca, Bristol Myers Squibb, Eli Lilly, Glaxo SmithKline, Janssen Cilag, Lundbeck, Merck, Pfizer, Sanofi Synthelabo, Servier, Solvay and Wyeth, and served as a consultant to Allergan, Astra Zeneca, Bioadvantex, Bionomics, Collaborative Medicinal Development, Eli Lilly, Grunbiotics, Glaxo SmithKline, Janssen Cilag, LivaNova, Lundbeck, Merck, Mylan, Otsuka, Pfizer, and Servier. KW received grant support from NHMRC. ST has received Grant/Research support from the NHMRC, State of Minnesota, TEVA pharmaceuticals, International Bipolar Foundation, and Brain and Research Foundation. The remaining authors declare that the research was conducted in the absence of any commercial or financial relationships that could be construed as a potential conflict of interest.

## Abbreviations

ACTH: adrenocorticotropic hormone
DBS: deep brain stimulation
dHIPP: dorsal hippocampus
ECL: enhanced chemiluminescence
Euth: euthanasia
FST: forced swim test
GSK3*β*: glycogen synthase kinase 3 beta
IACUC: Institutional Animal Care and Use Committee
IL: infralimbic cortex
mTOR: mammalian target of rapamycin
NAc: nucleus accumbens
PVDF: polyvinylidene fluoride
RiPA: radioimmunoprecipitation assay
SDS-PAGE: sodium dodecyl sulfate polyacrylamide gel electrophoresis
SPT: sucrose preference test
vHIPP: ventral hippocampus.
